# A Helquat-like Compound as a Potent Inhibitor of Flaviviral and Coronaviral Polymerases

**DOI:** 10.3390/molecules27061894

**Published:** 2022-03-15

**Authors:** Eva Konkolova, Kateřina Krejčová, Luděk Eyer, Jan Hodek, Michala Zgarbová, Andrea Fořtová, Michael Jirasek, Filip Teply, Paul E. Reyes-Gutierrez, Daniel Růžek, Jan Weber, Evzen Boura

**Affiliations:** 1 Institute of Organic Chemistry and Biochemistry, Academy of Sciences of the Czech Republic, v.v.i., Flemingovo nám. 2, 16610 Prague, Czech Republic; eva.konkolova@uochb.cas.cz (E.K.); krejcova@uochb.cas.cz (K.K.); jan.hodek@uochb.cas.cz (J.H.); michala.zgarbova@uochb.cas.cz (M.Z.); michal.jirasek@uochb.cas.cz (M.J.); filip.teply@uochb.cas.cz (F.T.); reyes.gutierrez@uochb.cas.cz (P.E.R.-G.); jan.weber@uochb.cas.cz (J.W.); 2 Laboratory of Emerging Viral Diseases, Veterinary Research Institute, Hudcova 296/70, 62100 Brno, Czech Republic; eyer@vri.cz (L.E.); andrea.fortova@vri.cz (A.F.); ruzekd@paru.cas.cz (D.R.); 3 Institute of Parasitology, Biology Centre of the Czech Academy of Sciences, Branišovská 1160/31, 37005 Ceske Budejovice, Czech Republic; 4 Department of Experimental Biology, Faculty of Science, Masaryk University, 62500 Brno, Czech Republic

**Keywords:** helquat-like compound, RNA-dependent RNA-polymerase, SARS-CoV-2, Flaviruses, antiviral agents

## Abstract

Positive-sense single-stranded RNA (+RNA) viruses have proven to be important pathogens that are able to threaten and deeply damage modern societies, as illustrated by the ongoing COVID-19 pandemic. Therefore, compounds active against most or many +RNA viruses are urgently needed. Here, we present PR673, a helquat-like compound that is able to inhibit the replication of SARS-CoV-2 and tick-borne encephalitis virus in cell culture. Using in vitro polymerase assays, we demonstrate that PR673 inhibits RNA synthesis by viral RNA-dependent RNA polymerases (RdRps). Our results illustrate that the development of broad-spectrum non-nucleoside inhibitors of RdRps is feasible.

## 1. Introduction

Flaviviruses (family *Flaviviridae*) and coronaviruses (family *Coronaviridae*) both belong among single-stranded positive-sense RNA (+RNA) viruses. Members of these viral families include important human pathogens, such as the yellow fever virus (YFV), Zika virus (ZIKV), tick-borne encephalitis virus (TBEV), West Nile virus (WNV), Middle East respiratory syndrome coronavirus (MERS-CoV), severe acute respiratory syndrome coronavirus (SARS-CoV), OC43 coronavirus (OC43-CoV) and the severe acute respiratory syndrome coronavirus 2 (SARS-CoV-2) [1,2,3,4]. Unfortunately, both families have proved that they hold pandemic potential, as demonstrated recently by the ZIKV, MERS-CoV and SARS-CoV outbreaks [5,6,7,8]. Nonetheless, the ZIKV, MERS-CoV and SARS-CoV viruses were contained. It was the SARS-CoV-2 that revealed the full potential of +RNA viruses to cause harm to humans. According to the WHO (https://covid19.who.int/, accessed on 20 February 2022), the COVID-19 pandemic has already claimed 6 million lives worldwide at the beginning of March 2022.

Small molecule-based antiviral treatments are urgently needed. The first FDA-approved small molecule to be used against COVID-19 was remdesivir [9], and recently orally available drugs molnupiravir and Paxlovid were approved in several countries as well. Molnupiravir targets the RNA-dependent RNA polymerase (RdRp), a key enzyme in the replication of RNA viruses [10]. The primary target of Paxlovid is the SARS-CoV-2 main protease, which is involved in the processing of the coronaviral polyprotein [11]. Importantly, new SARS-CoV-2 variants, such as omicron, that partially escape vaccine-induced immunity are sensitive to these compounds [12]. In addition, other coronaviral enzymes, including exonuclease, endonuclease, helicase and methyltransferase, have recently been biochemically and structurally described [13,14,15,16,17], and their inhibitors have been reported [18,19,20,21]; however, none of these compounds have been developed enough to enter clinical trials. Furthermore, often, the simultaneous administration of several compounds is necessary to efficiently combat a virus and to prevent the development of escape mutants, as illustrated by the highly efficient highly active antiretroviral therapy (HAART) against the HIV virus [22].

**Figure 1:**
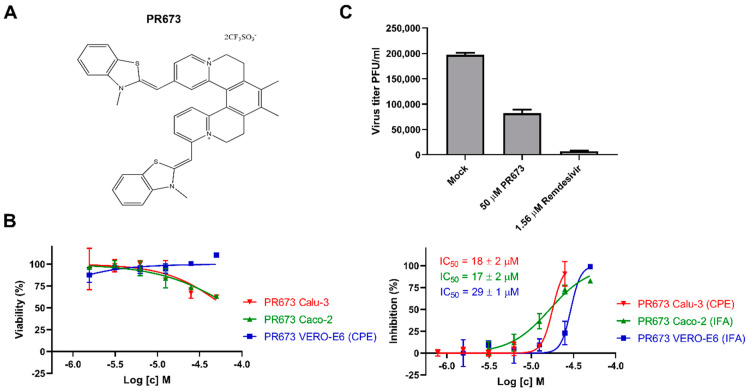
Inhibitory activity of PR673 against SARS-CoV-2. (**A**) The chemical structure of PR673. (**B**) Left panels: dependence of viability of VERO-E6, Calu-3 and Caco-2 cells on PR673 concentration; right panels: dependence of inhibition of SARS-CoV-2 in VERO-E6, Calu-3 and Caco-2 cells on PR673 concentration. (**C**) Comparison of PR673 and remdesivir in SARS-CoV-2 yield reduction assay. The VERO-E6 cells were incubated with PR673, remdesivir and a mock followed by SARS-CoV-2 infection at MOI 0.04 for 3 days, and the virus yield was determined by plaque assay in VERO-E6 cells.

Here, we present compound PR673 bearing a rather unusual helquat-like chemical structure (Figure 1A) that was discovered while screening against the SARS-CoV-2 virus, but is actually more active against flaviviruses. We use in vitro polymerase assays to show that this compound interferes with RNA syntheses performed by the viral RdRps.

## 2. Results

### 2.1. Identification of PR673

We screened the IOCB library [23] using a phenotypic assay against SARS-CoV-2. Briefly, the IOCB-library compounds were screened at a 64 µM concentration in triplicates in VERO-E6 cells against SARS-CoV-2, and the inhibition of the virus-induced cytopathic effect (CPE) was monitored. We identified an interesting helquat-like compound (PR673) (Figure 1A) that inhibited the replication of the virus in VERO-E6 cells. This activity was further verified in VERO-E6 and Caco-2 cells using SARS-CoV-2 nucleoprotein expression detected by the immunofluorescence assay (IFA) (Figure 1B, Supplementary Figure S1), which we could also confirm using the protection of cells from the virus-induced cytopathic effect (CPE) in Calu-3 cells (Figure 1B). PR673 inhibited SARS-CoV-2 with an EC_50_ value of 29 µM in VERO-E6 cells (Figure 1B) and yielded a 58% reduction in viral plaques at 50 µM (Figure 1C), while exhibiting no cell toxicity in Vero E6 cells (Figure 1B). The compound effectively inhibited virus-induced CPE in Calu-3 cells and virus replication in Caco-2 cells, with EC_50_ values of 18 µM and 17 µM, respectively. We observed mild cytotoxicity at the highest used concentration for Caco-2 and Calu-3 cell lines, but the CC_50_ of PR673 for both cell lines was above 50 µM (Figure 1B). Nonetheless, it must be noted that PR673 was much less active than the COVID-19 drug remdesivir (Figure 1C).

### 2.2. PR673 Inhibits the Coronaviral RdRp

**Figure 2:**
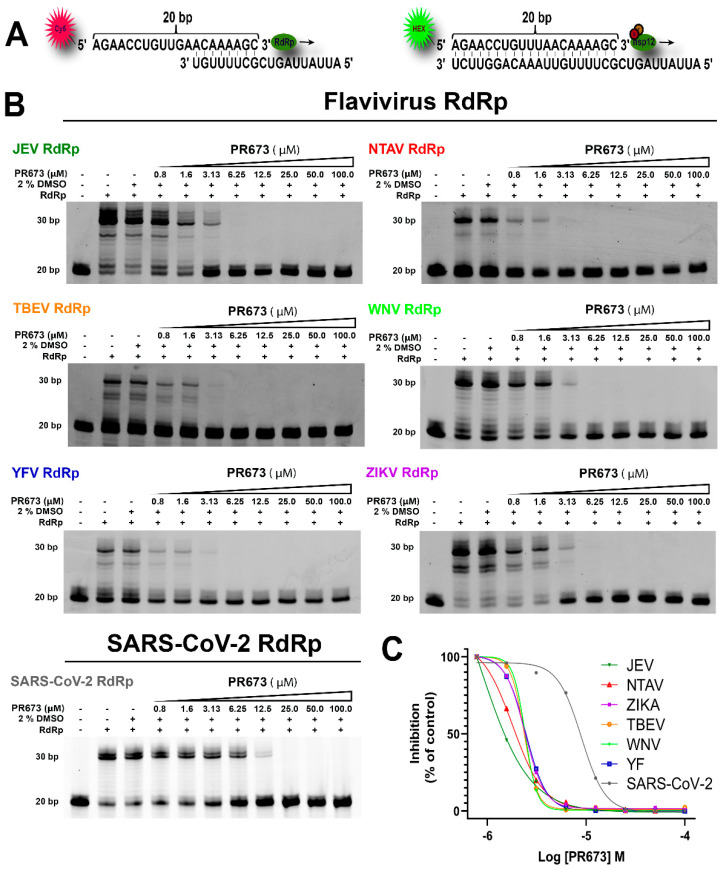
Analysis of PR673 inhibitory activity against various RdRps using a primer extension assay. (**A**) The RNA primer and RNA templates used in this assay. The RNA primer contains a fluorescent label at the 5′ end (Cy5 or Hex for flaviviral RdRp, or SARS-CoV-2 RdRp, respectively). The arrow indicates the direction of primer extension. (**B**) Serial dilutions of PR673 (in µM), as indicated on the top of the gel, and a constant concentration of the polymerase (20 nM) and template/primer (10 nM) were used in the assay. The reactions were initiated by adding 10 µM NTPs. The reactions continued at 33 °C for 1 h; then, the reactions were stopped by the addition of stop buffer, and the products were separated on denaturing polyacrylamide gels. (**C**) The percentages of inhibition (against control) in panel B were plotted against the logarithm of concentrations of PR673; the results were fitted to sigmoidal dose–response curves.

We next aimed to discover the molecular target of this compound. Based on the chemical structure, we speculated that the RdRp is the target of PR673. The SARS-CoV-2 RdRp is a heterotrimeric protein complex composed of nsp7, nsp8 and nsp12. We prepared this RdRp recombinantly, as reported before [21], and measured the inhibitory activity of PR673. Indeed, it targeted the SARS-CoV-2 RdRp: the synthesis of RNA was totally blocked at a PR673 concentration above 12.5 µM (Figure 2, SARS-CoV-2 panel).

### 2.3. PR673 Inhibitory Activity against Flaviviral RdRps

**Figure 3:**
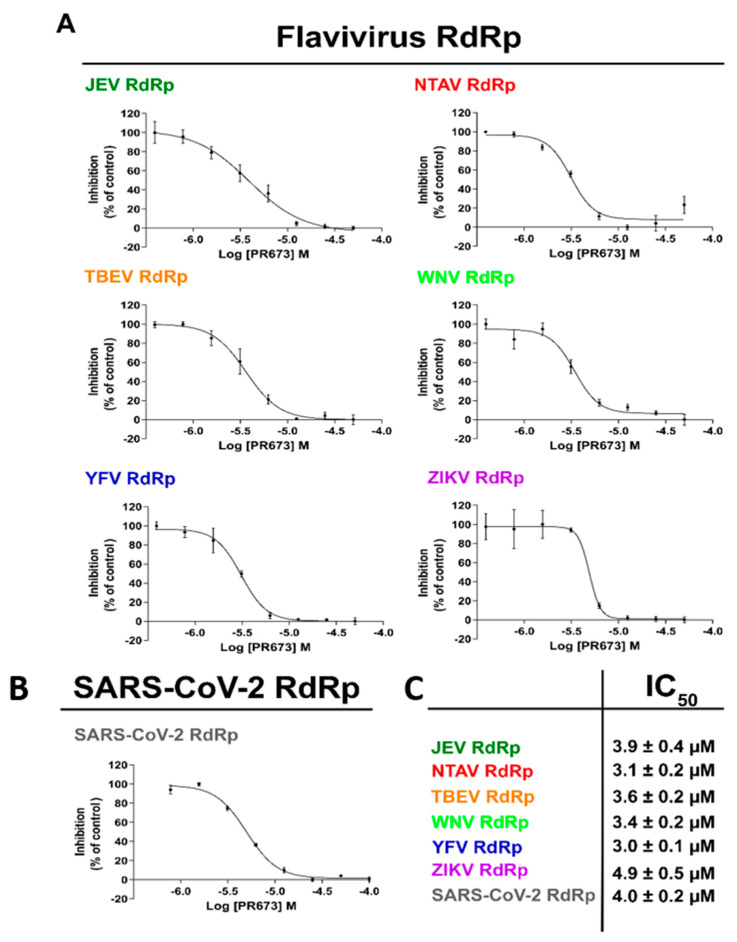
Measurement of the IC_50_ values of PR673. The IC_50_ values were established for each tested polymerase with PR673 using radioactively labeled elongation products. (**A**) The RNA hairpin used in this assay. The arrow indicates the direction of primer extension. (**B**) The percentages of inhibition (against control) were plotted against the logarithm of concentrations of PR673, and the results were fitted to sigmoidal dose–response curves. Error bars represent the standard error of three independent measurements. (**C**) The table of IC_50_ values. The IC_50_ values were extrapolated from LogIC_50_ according to the GraphPad algorithm.

Further, we sought to decipher whether PR673 inhibited other viral RdRps. We chose several members of the *Flaviviridae* family, namely the Japanese encephalitis virus (JEV), Ntaya virus (NTAV), tick-borne encephalitis virus (TBEV), yellow fever virus (YFV) and Zika virus (ZIKV), and we prepared their polymerases recombinantly. In each case, we observed a strong inhibition of RNA synthesis in low micromolar concentrations of PR673 (Figure 2 and Figure 3). 

**Figure 4:**
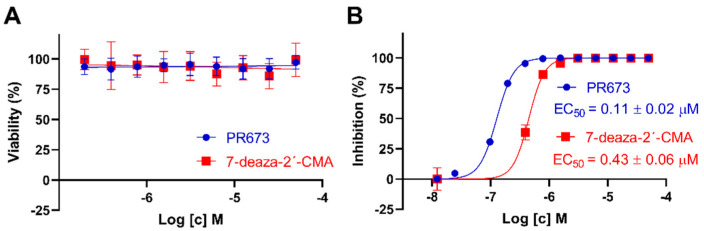
Cytotoxicity and anti-TBEV activity of PR673 in PS cells. (**A**) Cytotoxicity of PR673 for PS cells expressed as percentage of cell viability at the indicated drug concentrations. The cells were seeded in 96-well plates for 24 h, then treated with the compounds and incubated for 48 h. Cell viabilities were measured by Cell Counting Kit-8. (**B**) Anti-TBEV activity of PR673 in PS cells. The cell monolayers were treated with the compounds (0 to 50 µM) and infected simultaneously with TBEV (Hypr) at MOI of 0.1. The infected cells were then incubated for 48 h, after which, cell media were collected and viral titers determined using a plaque assay. The obtained titers were used to construct dose-dependent inhibition curves (as indicated) and to calculate EC_50_ values. 7-deaza-2′-CMA (in the same concentration range) was used as a positive control.

We then established IC_50_ values for each of the aforementioned RdRp enzymes using a radioactive assay because it is more sensitive and accurate than alternative methods. The reaction mixture contained the viral RdRp, the hairpin-containing RNA as the template and radioactively labeled ATP ([α-^32^P]-ATP). Our results showed that PR673 inhibits all of the tested polymerases, with IC_50_ values ranging from 3.0 ± 0.1 µM to 4.9 ± 0.5 µM (Figure 4). The yellow fever virus RdRp was inhibited the most and the Zika RdRp the least; however, the observed differences were rather low.

### 2.4. PR673 Inhibits Replication of the TBEV in Cell Culture

Since we observed a strong inhibition of all tested flaviviral RdRps in vitro, we decided to test whether PR673 could inhibit a flavivirus in cell culture. We used the TBEV (strain Hypr) for which we have well established assays [24,25]. We first tested the cytotoxicity of PR673 in porcine kidney stable cells (PS cells) that are widely used for TBEV multiplication, anti-TBEV assays and TBEV-based plaque assays [26]. We also used 7-deaza-2′-C-methyladenosine (7-deaza-2′-CMA) as a positive control because this compound is a well-established inhibitor of the TBEV RdRp [25]. The cytotoxicity was evaluated in a concentration range of 0 to 50 µM; both compounds appeared as non-cytotoxic for PS cells up to 50 µM when incubated with the cells for 48 h. Similarly to 7-deaza-2′-CMA, the CC_50_ values of PR673 were estimated to be >50 µM (Figure 4A).

Next, we tested the anti-TBEV activity of PR673 in PS cells and compared its anti-TBEV activity with that of 7-deaza-2′-CMA. PR673 exerted a dose-dependent anti-TBEV effect, with an EC_50_ value of 0.11 µM. The complete inhibition of TBEV replication was observed at a compound concentration of 0.4 µM. The anti-TBEV activity of PR673 was almost four-fold higher in comparison to 7-deaza-2′-CMA (Figure 4B).

## 3. Discussion

More small molecules active against SARS-CoV-2 are needed. Two compounds targeting the RdRp (remdesivir and molnupiravir) and the 3C-like protease inhibitor nirmatrelvir (sold under the name Paxlovid) are already available as human medicines. In addition, inhibitors of other coronaviral enzymes, such as the helicase [27,28], endo- and exonuclease [29,30] or the methyltransferases [18,19,31], have been actively developed. However, the goal is to develop compounds that would be active against multiple viruses or even multiple viral families. These so-called broad-spectrum antivirals could be a powerful weapon to combat future pandemics. One such example is remdesivir. It was originally discovered by Gilead when screening for compounds active against the respiratory syncytial virus, and was later both developed to combat the Ebola outbreak in 2014 and repurposed against SARS-CoV-2 [32,33,34]. Recently, we showed that it can also effectively inhibit flaviviral RdRps [35]. Remdesivir is a nucleotide analog that is metabolized into remdesivir triphosphate; upon incorporation into RNA, it acts as a delayed chain terminator. In fact, nucleoside analogs can be divided into three classes: (*i*) mutagenic nucleosides, such as ribavirin, that are able to cause mutational catastrophe [36]; (*ii*) obligate chain terminators that usually lack the ribosyl C3′-hydroxyl group [37]; and (*iii*) delayed chain terminators, such as remdesivir [38]. Our experiments with PR673 demonstrate that it acts as a pseudo-obligate chain terminator. It can clearly stop the synthesis of RNA in vitro by recombinant RdRps (Figure 2), which explains its effect on the replication of SARS-CoV-2 and TBEV (Figure 1 and Figure 4). However, obligate chain terminators are incorporated into RNA, and their chemical nature prevents the incorporation of another nucleoside [39], which is clearly not the case for PR673. It could possibly compete for one of the RNA binding sites that are present and relatively conserved at viral RdRps, such as the entry site or the exit tunnel [40,41,42]. We cannot rule out the possibility that PR673 is an allosteric inhibitor; however, this is not probable because it is active on relatively distant RdRps, where an allosteric site is unlikely to be conserved. In any case, PR673 illustrates that the development of non-nucleoside antivirals active against a broad spectrum of viruses is feasible.

## 4. Material and Methods

### 4.1. Anti-SARS-CoV-2 Activity Determination Using Immunofluorescence Assay

Anti-SARS-CoV-2 activity was tested in VERO-E6 (ATCC CRL-1586) and Caco-2 cells (ATCC HTB-37) using immunofluorescence assay. VERO-E6 cells were seeded one day before experiment in DMEM medium with 10% FBS, 100 U of penicillin/mL and 100 µg of streptomycin/mL (all Merck KGaA, Darmstadt, Germany) in 96-well plate. Day after, two-fold serial dilution of compound was added to the cells in triplicate in complete DMEM medium with 2% FBS. After one hour, cells were infected with SARS-CoV-2 (hCoV-19/Czech Republic/NRL_6632_2/2020, multiplicity of infection MOI = 0.02) and incubated for 3 days in 5% CO_2_ at 37 °C. After incubation, medium was removed, cells were fixed using 4% paraformaldehyde (PFA), washed 3× with PBS, permeabilized with 0.2% Triton-X100 for 5 min at room temperature and incubated for 2 h with anti-SARS-CoV-2 antibody (mouse monoclonal anti-nucleoprotein IgG, Institute of Molecular Genetics, Czech Republic). Subsequently, cells were washed 3× with PBS and incubated for 1.5 h with 1:250 dilution of Cy3-labeled donkey anti-mouse IgG (Jackson ImmunoResearch, Cambridgeshire) at RT, and fluorescent foci were visualized using a fluorescence microscope (Olympus IX 81, Hamburg, Germany). Cellular DNA was labeled with DAPI (4′,6-diamidino-2-phenylindole) (Merck KGaA, Darmstadt, Germany) nucleic acid stain. Cells were incubated with DAPI (0.1 µg/mL) for 10 min and subsequently washed with 1× PBS. Immunofluorescence assay in Caco-2 cells was performed similarly as above, with following changes. The MOI of SARS-CoV-2 was 0.002, permeabilization was performed with methanol at −20 °C for ten minutes and, as a secondary antibody, the 1:200 dilution of FITC-labelled goat anti-mouse IgG (Jackson ImmunoResearch) was used. The fluorescent images of both cell lines were analyzed by ImageJ (NIH) and the compound concentration required to reduce fluorescence by 50% (EC_50_) was calculated using nonlinear regression analysis with GraphPad Prism software.

### 4.2. Anti-SARS-CoV-2 Activity Determination Using Cytopathic Effect-Based Assay

For CPE-based assay, two-fold serial dilutions of compounds were added in triplicate in a 96-well plate with Calu-3 cells (ATCC HTB-55) seeded day before in DMEM medium with 10% FBS, 100 U of penicillin/mL and 100 µg of streptomycin/mL. After 1 h incubation, SARS-CoV-2 was added at MOI 0.04. Following a three-day incubation at 37 °C in 5% CO_2_ the cell viability was evaluated by XTT cell viability assay. Four hours after addition of XTT solution, the absorbance was measured using EnVision plate reader (PerkinElmer) and the compound concentrations required to reduce viral cytopathic effect by 50% (EC_50_) were calculated using nonlinear regression analysis using GraphPad Prism software.

### 4.3. Cytotoxicity Determination in SARS-CoV-2 Assays 

Cytotoxicity was evaluated by incubating the same two-fold serial dilutions of compound as in antiviral assays with VERO-E6, Caco-2 and Calu-3 cells. After three days’ incubation at 37 °C in 5% CO_2_, the cell viability was determined by addition of 50:1 mixture of XTT labeling reagent (1 mg/mL) and PMS electron-coupling reagent (0.383 mg/mL) (both Merck KGaA, Darmstadt, Germany), and the compound concentrations resulting in 50% reduction in viability (CC_50_) were calculated as above in the antiviral activity determination using CPE-based assay.

### 4.4. SARS-CoV-2 Yield Reduction Assay

The VERO-E6 cells were incubated with and without a tested compound (at different concentrations) for 1 h, followed by SARS-CoV-2 infection at MOI 0.04 for 3 days at 37 °C in 5% CO_2_. Virus yield was determined by a plaque assay in a 24-well plate. Briefly, 100 µL of the supernatant was added to VERO-E6 cell monolayer, incubated for 4 h at 37 °C in 5% CO_2_ and overlaid with 3% carboxymethyl cellulose. After a 5-day incubation, the cells were fixed and stained with Naphthalene black and the plaques were counted.

### 4.5. Anti-TBEV Studies

TBEV strain Hypr, a representative of the European TBEV subtype, was provided by the Collection of Arboviruses, Institute of Parasitology, Biology Centre of the Czech Academy of Sciences, Ceske Budejovice, Czech Republic (http://www.arboviruscollection.cz/index.php?lang=en, accessed on 20 February 2022). Porcine kidney stable (PS) cells [26] were cultured in Leibovitz (L-15) medium and supplemented with 3% newborn calf serum, 100 U/mL penicillin, 100 µg/mL streptomycin and 1% glutamine (Sigma-Aldrich KGaA, Darmstadt, Germany). PS cells were cultivated at 37 °C under a normal atmosphere (without CO_2_ supplementation).

To determine the cytotoxicity of PR673, PS cells were seeded in 96-well microtitration plates (2 × 10^4^ cells per well) and incubated for 24 h at 37 °C. After incubation, PR673 was added to the cells (0 to 50 µM). 7-deaza-2′-CMA (Carbosynth, Compton, UK) in the same concentration range was used as a positive control. Then, the treated cells were cultivated for 48 h at 37 °C. The cytotoxicity measured in terms of cell viability was determined with Cell Counting Kit-8 (Dojindo Molecular Technologies) according to manufacturer’s instructions. The respective concentrations of each compound that reduced cell viability by 50% (CC_50_ values) were determined. The experiment was performed in triplicate.

TBEV titer reduction assays were performed to determine anti-TBEV activity of PR673 in the PS cell culture. The cells were seeded in 96-well plates (2 × 10^4^ cells per well) and incubated for 24 h at 37 °C to form a confluent monolayer. Subsequently, the cells were infected with TBEV (multiplication of infection of 0.1), simultaneously treated with PR673 at concentrations of 0 to 50 µM and incubated for 48 h at 37 °C. Following incubation, media were collected and viral titers determined by plaque assay [25] to construct dose-dependent inhibition curves and to estimate 50% effective concentration (EC_50_) values. Similarly to the cytotoxicity assays, the experiment was performed in triplicate and the 7-deaza-2′-CMA was used as a positive control.

### 4.6. Protein Expression and Purification

NS5 proteins and SARS-CoV-2 nsp12, nsp8 and nsp7 were expressed with appropriate purification and folding tags, as detailed in Supplementary Table S1 and as described before [21,35,39]. Briefly, all of the proteins were expressed in bacterial cells, except for the full length nsp12 protein, which was expressed in insect cells. Other genes were expressed in *E. coli* (BL-21 CodonPlus (DE3)-RIL). Transformed cells were grown to an optimal OD_600_ in LB medium at 37 °C; then, the protein expression was induced by addition of IPTG to 0.4 mM and the cells were grown at 18 °C for 16 h. The recombinant proteins were purified using Ni^2+^ affinity chromatography followed by tag cleavage (when appropriate), and further purified using size exclusion chromatography.

### 4.7. Primer Extension Polymerase Activity Assay

The polymerase activity was determined in a primer extension reaction using a fluorescently labeled primer 1 (P1: 5′-Cy5-AGAACCUGUUGAACAAAAGC-3′) or primer 2 (P2: 5′-HEX-AGAACCUGUUUAACAAAAGC-3′) and an RNA template 1 (T1: 5′-AUUAUUAGCUGCUUUUGU-3′) or template 2 (T2: 5′-AUUAUUAGCUGCUUUUGUUAAACAGGUUCU-3′). All primers were synthesized by Sigma-Aldrich. 

The polymerase activity assay was performed in a total volume of 10 μL of reaction mixture containing reaction buffer, NTPs, template/primer, the viral polymerase and 0.01U RNasin (New England BioLabs Int.). The exact composition of each reaction mixture was optimized for each polymerase, as detailed in Supplementary Table S2. The reactions were incubated for 1 h at 30 °C for SARS-CoV-2 RdRp or 1 h at 33 °C for flaviviral RdRp, respectively. After incubation, reactions were stopped by adding 20 μL of stop buffer (80% formamide, 50 mM EDTA), samples were denatured at 95 °C for 10 min and primer extension products were separated on a 20% denaturing polyacrylamide gel (8 M urea, 1× TBE, 20% acryl amide (19:1)). After electrophoresis, gels were scanned on Amersham Typhoon 5 Biomolecular Imager (GE Healthcare) and analyzed by ImageQuant TL8.2 (Cytiva). 

### 4.8. In Vitro Determination of IC_50_

IC_50_ values were determined in a similar polymerase activity assay as above but using radioactive labeling. The assay was performed in a total volume of 20 µL reaction mixture (Supplementary Table S2), but, as template, we used an RNA oligo containing a hairpin 5′-25U-HP-3′(5′-UUUUUUUUUUUUUUUUUUUUUUUUUAACAGGUUCUAGAACCUGUU-3′) and NTPs were replaced by 0.01 µCi/µL [α-^32^P]-ATP. After incubation, 5 µL of reaction mixtures was spotted on anion exchange cellulose filter paper (Whatman^TM^ Grade DE81 DEAE cellulose paper; GE Healthcare) in triplicate. The Whatman filter was then dried, subsequently washed by 0.125 mM Na_2_HPO_4_, water and ethanol and dried again. Dry filter paper was then analyzed using phosphorimaging. The plate was scanned on Amersham Typhoon 5 Biomolecular Imager (GE Healthcare), the products were quantified with Image Studio Lite (LI-COR) and data were analyzed using GraphPad version 6 (GraphPad Prism version 6, GraphPad Software, San Diego, CA, USA).

## Data Availability

Not applicable.

## Supplementary Materials

The following supporting information can be downloaded at: https://www.mdpi.com/article/10.3390/molecules27061894/s1, Figure S1: Immunofluorescence microscopy analysis of SARS-CoV-2 nucleoprotein expression after addition of 50 μM, 25 μM, and 12.5 μM of PR673; Figure S2: Synthesis of PR673 by a condensation reaction between the helquat MJB and methylbenzothiazole PR739; Table S1: Summery of viral polymerases list of polymerases used in this study; Table S2: Exact composition of each polymerase reaction mixture.

## Sample Availability

Samples of the compounds are available from the corresponding author upon request.
