# Supplementary material for: A Helquat-like Compound as a Potent Inhibitor of Flaviviral and Coronaviral Polymerases
Source: Molecules. 2022 Mar 15;27(6):1894. doi: 10.3390/molecules27061894 (PMC8953834; doi:10.3390/molecules27061894)
Supplement: Supplementary file 1 [file molecules-27-01894-s001.zip › molecules-1627944-supplementary.pdf]

Article

A Helquat-like Compound as a Potent Inhibitor of Flaviviral and Coronaviral Polymerases

Eva Konkolova [1,†], Kateřina Krejčová [1,†], Luděk Eyer [2,†], Jan Hodek [1], Michala Zgarbová [1], Andrea Fořtová [2], Michael Jirasek [1], Filip Teply [1], Paul E. Reyes-Gutierrez [1], Daniel Růžek [2,3,4], Jan Weber [1] and Evzen Boura [1,*]

[1] Institute of Organic Chemistry and Biochemistry, Academy of Sciences of the Czech Republic, v.v.i, Flemingovo nám. 2, 16610 Prague, Czech Republic; eva.konkolova@uochb.cas.cz (E.K.); krejcova@uochb.cas.cz (K.K.); jan.hodek@uochb.cas.cz (J.H.); michala.zgarbova@uochb.cas.cz (M.Z.); michal.jirasek@uochb.cas.cz (M.J.); filip.teply@uochb.cas.cz (F.T.); reyes.gutierrez@uochb.cas.cz (P.E.R.-G.); jan.weber@uochb.cas.cz (J.W.)

[2] Laboratory of Emerging Viral Diseases, Veterinary Research Institute, Hudcova 296/70, 62100 Brno, Czech Republic; eyer@vri.cz (L.E.); andrea.fortova@vri.cz (A.F.); ruzekd@paru.cas.cz (D.R.)

[3] Institute of Parasitology, Biology Centre of the Czech Academy of Sciences, Branišovská 1160/31, 37005 Ceske Budejovice, Czech Republic

[4] Department of Experimental Biology, Faculty of Science, Masaryk University, 62500 Brno, Czech Republic

* Correspondence: boura@uochb.cas.cz

† These authors contributed equally to this work.

Table S1. Summary of viral polymerases list of polymerases used in this study.

**JEV**		NP_775674.1	pET28b	N- terminal His_{6x}-TEV
**NTAV**		YP_006846328.2	pET28b	N- terminal His_{6x}-TEV
**TBEV**		AKP16371.1	pET26-Ubq	N- terminal Ubiquitin and C-terminal His_{6x}
**ZIKV**		APP91864.1	pSUMO	N- terminal His_{8x}-SUMO
**WNV**		YP_001527887.1	pET28b	N- terminal His_{6x}-TEV
**YFV**		NP_041726.1	pSUMO	N- terminal His_{8x}-SUMO
**SARS-CoV-2**	nsp12	YP_009725307	pAceBac	C- terminal His_{6x}
	nsp7	YP_009725303	pHGT2	N- terminal His_{6x}-GB1-TEV
	nsp8	YP_009725304	pHGT2	-

Table S2. Exact composition of each polymerase reaction mixture.

	**Flavivirus RdRp**	**SARS-CoV-2 RdRp**
**Buffer**	5 mM Tris 7.4,<br>10 mM DTT,<br>2 mM $MnCl_2$,<br>0.5 % Triton X-100,<br>10% glycerol	10 mM Tris pH 8.0,<br>1 mM β-mercaptoethanol,<br>2 mM $MgCl_2$,<br>10 mM KCl,
**NTPs [µM]**	10	10
**T/P [µM]**	**T1/P1** – 0.01	**T2/P2** – 0.5
**Polymerase [µM]**	0.02	1 (nsp12)<br>3 (nsp7/nsp8 complex)

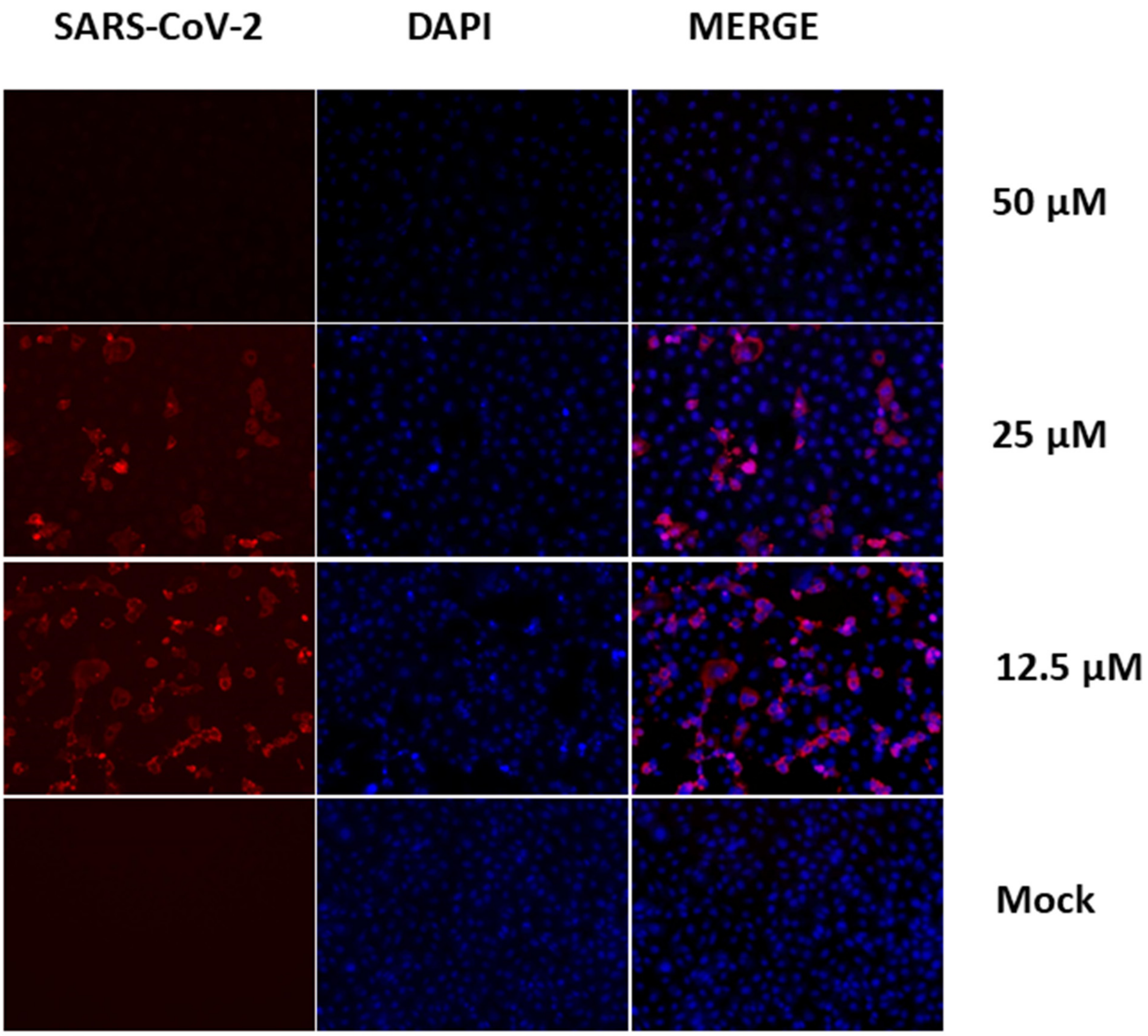

Figure S1. Immunofluorescence microscopy analysis of SARS-CoV-2 nucleoprotein expression after addition of 50 µM, 25 µM, and 12.5 µM of PR673. VERO-E6 cells with and without (Mock) PR673 were infected with SARS-CoV-2 with MOI = 0.02, fixed 3 days post infection, stained with specific SARS-CoV-2 anti-nucleoprotein antibody followed by Cy3-conjugated anti-mouse antibody (red) and counterstained with DAPI (blue).

Synthesis of PR673

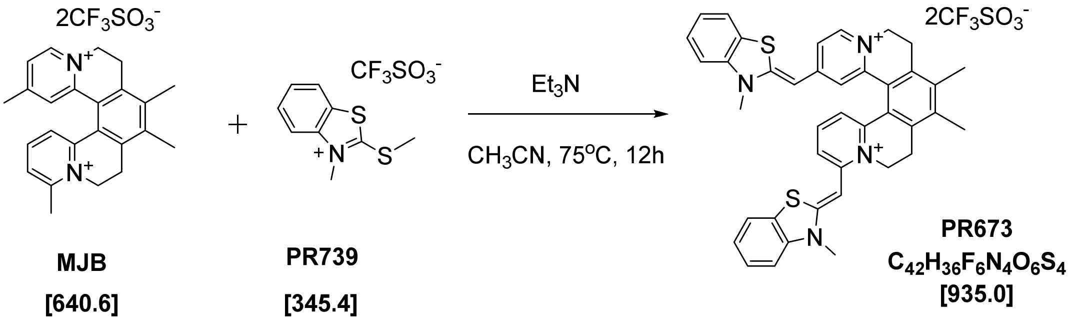

Figure S2. Synthesis of **PR673** by a condensation reaction between the helquat **MJB** and methylbenzothiazole **PR739**.

Starting helquat **MJB** (1.0 g, 1.0 equiv, 1.56 mmol), **PR379** (4.3 g, 8.0 equiv, 12.5 mmol), Et_3N (1.26 g, 1.74 mL, 8.0 equiv, 12.5 mmol) and dry CH_3CN (50 mL) were placed into a 100 mL flask and the resulting mixture was stirred under argon for 12 h at 75°C. Progress of the reaction was monitored by thin layer chromatography (mobile phase Stoddart's magic mixture, MeOH: 2 M NH_4Cl: $MeNO_2$ = 7: 2: 1). Crude product was transferred to 50 mL centrifuge tubes (6 tubes with 8.3 mL of reaction crude each one) and precipitated from reaction mixture by addition of Et_2O (35 mL to each tube). The resulting suspensions were centrifuged and supernatants were removed. Residues were dissolved in a minimum amount of CH_3CN/piperidine solution (60:40, 5 mL added to each tube), afterward Et_2O (35 mL) was added to each tube. Precipitates were centrifuged. This reprecipitation was repeated 6 times more. Centrifuged solids were finally washed with Et_2O. The resulting suspension was centrifuged and Et_2O was removed. The solids were dried under vacuum to get pure **PR673** as a deep red solid in 77% yield (1.12 g, 1.2 mmol).

^{1}H NMR (400 MHz, DMSO) δ 8.46 (d, *J* = 7.0 Hz, 1H), 7.76 (dd, *J* = 10.6, 6.1 Hz, 3H), 7.65 (d, *J* = 8.3 Hz, 1H), 7.61 – 7.50 (m, 3H), 7.44 (d, *J* = 8.3 Hz, 1H), 7.29 (dtd, *J* = 15.7, 8.1, 7.5, 2.8 Hz, 3H), 6.93 (d, *J* = 2.3 Hz, 1H), 6.85 (t, *J* = 7.7 Hz, 1H), 6.37 (s, 1H), 6.09 (s, 1H), 5.23 (d, *J* = 13.9 Hz, 1H), 4.71 (d, *J* = 12.7 Hz, 1H), 4.50 (t, *J* = 13.4 Hz, 1H), 4.36 (t, *J* = 13.6 Hz, 1H), 3.97 (s, 3H), 3.51 – 3.26 (m, 4H), 3.02 (t, *J* = 16.3 Hz, 2H), 2.42 (d, *J* = 10.6 Hz, 6H). ^{13}C NMR (101 MHz, DMSO) δ 157.85, 155.73, 152.70, 148.87, 146.69, 144.87, 141.22, 140.93, 140.26, 139.60, 138.64, 138.20, 138.13, 137.71, 127.72, 127.63, 125.50, 125.04, 123.66, 122.83, 122.64, 122.42, 122.40, 122.28, 122.21, 121.88, 120.27, 119.08, 111.79, 89.48, 83.10, 51.08, 47.62, 33.17, 32.76, 25.91, 25.70, 16.05. MS (ESI) m/z (%): 785.2 (TfO^-, 40), 318.1 (- 2TfO^-, z=2,100). HRMS (ESI) m/z: [(M-2TfO^-)] ($C_{40}H_{36}N_4S_2^{2+}$) calc.: 318.11852 (z=2), found: 318.11856 (z=2).

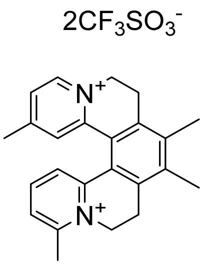

MJB

^{1}H NMR (400 MHz, DMSO) δ 9.00 (d, *J* = 6.4 Hz, 1H), 8.09 (t, *J* = 8.0 Hz, 1H), 7.97 (dd, *J* = 8.3, 1.5 Hz, 1H), 7.92 (dd, *J* = 7.8, 1.5 Hz, 1H), 7.83 (dd, *J* = 6.4, 1.9 Hz, 1H), 7.79 (d, *J* = 1.8 Hz, 1H), 5.14 (dd, *J* = 13.9, 3.8 Hz, 1H), 5.02 – 4.91 (m, 1H), 4.70 (t, *J* = 14.0 Hz, 1H), 4.37 (td, *J* = 14.3, 3.6 Hz, 1H), 3.53 – 3.37 (m, 2H), 3.08-2.97 (m, 5H), 2.40 (d, *J* = 2.8 Hz, 6H), 2.31 (s, 3H). ^{13}C NMR (101 MHz, DMSO) δ 157.02, 155.07, 147.45, 146.19, 144.16, 142.62, 139.25, 138.95, 129.72, 128.05, 127.83, 126.22, 124.66, 53.11, 48.55, 25.15, 24.90, 21.00, 16.19, 16.15. MS (ESI) m/z (%): 491.2 (TfO^-, 10), 341.2 (-2TfO^-,100). HRMS (ESI) m/z: [(M-2TfO^-)] ($C_{24}H_{26}N_2^{2+}$) calc.: 171.10425 (z=2), found: 171.10423 (z=2).

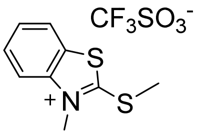

PR739

2-(Methylthio)benzothiazole (5.0 g, 1.0 equiv, 27.6 mmol) was dissolved in dry DCM (100 mL). Then, methyl trifluoromethanesulfonate (5.43 g, 3.63 ml, 1.2 equiv, 33.1 mmol) was added slowly and reaction mixture was stirred under argon for 12 h at RT. Crude product was transferred to 50 mL centrifuge tubes (3 tubes with 33.3 mL of reaction crude each one). The resulting suspensions were centrifuged and DCM was removed. Residues were washed with Et_2O (35 mL) by sonication. Precipitates were centrifuged and Et_2O was removed. This process was repeated 4 times more. The solids were dried under vacuum to get pure **PR739** as a white off solid in 79% yield (7.5 g, 21.8 mmol).

^{1}H NMR (400 MHz, DMSO) δ 8.38 (dd, *J* = 8.2, 1.3 Hz, 1H), 8.18 (d, *J* = 8.4 Hz, 1H), 7.84 (dd, *J* = 8.5, 7.3 Hz, 1H), 7.72 (dd, *J* = 8.5, 7.3 Hz, 1H), 4.11 (s, 3H), 3.12 (d, *J* = 0.8 Hz, 3H). ^{13}C NMR (101 MHz, DMSO) δ 181.35, 142.53, 129.18, 128.28, 127.03, 123.94, 115.70, 36.42, 18.02. MS (ESI) m/z (%): 196.0 (- TfO^-, 25), 181.0 (- TfO^-, - Me, 100). HRMS (ESI) m/z: [(M-TfO^-)] ($C_9H_{10}NS_2^+$) calc.: 196.02492, found: 196.02497.

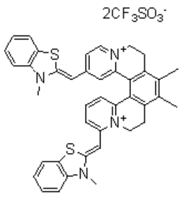
2CF3SO3-
S
N
N+
N+
S
N

PR673

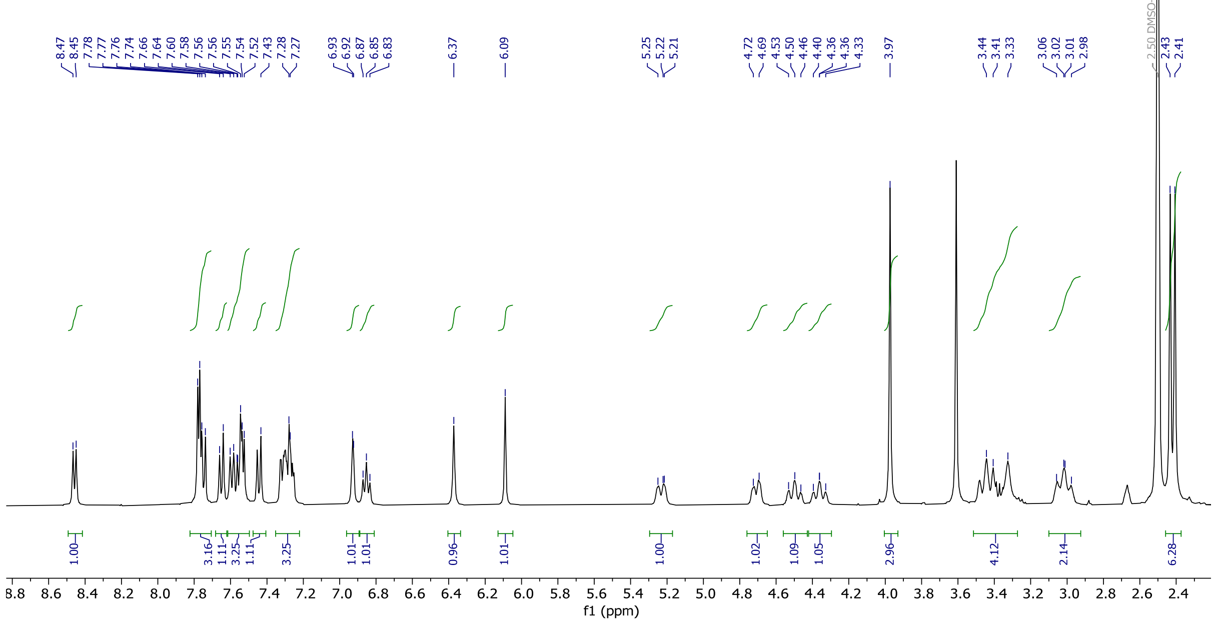
8.47
8.45
7.78
7.77
7.76
7.74
7.66
7.64
7.60
7.58
7.56
7.56
7.55
7.54
7.52
7.43
7.28
7.27
6.93
6.92
6.87
6.85
6.83
6.37
6.09
5.25
5.22
5.21
4.72
4.69
4.53
4.50
4.46
4.40
4.36
4.36
4.33
3.97
3.44
3.41
3.33
3.06
3.02
3.01
2.98
2.50 DMSO-d6
2.43
2.41
1.00
3.16
1.11
3.25
1.11
3.25
1.01
1.01
0.96
1.01
1.00
1.02
1.09
1.05
2.96
4.12
2.14
6.28
8.8 8.6 8.4 8.2 8.0 7.8 7.6 7.4 7.2 7.0 6.8 6.6 6.4 6.2 6.0 5.8 5.6 5.4 5.2 5.0 4.8 4.6 4.4 4.2 4.0 3.8 3.6 3.4 3.2 3.0 2.8 2.6 2.4
f1 (ppm)

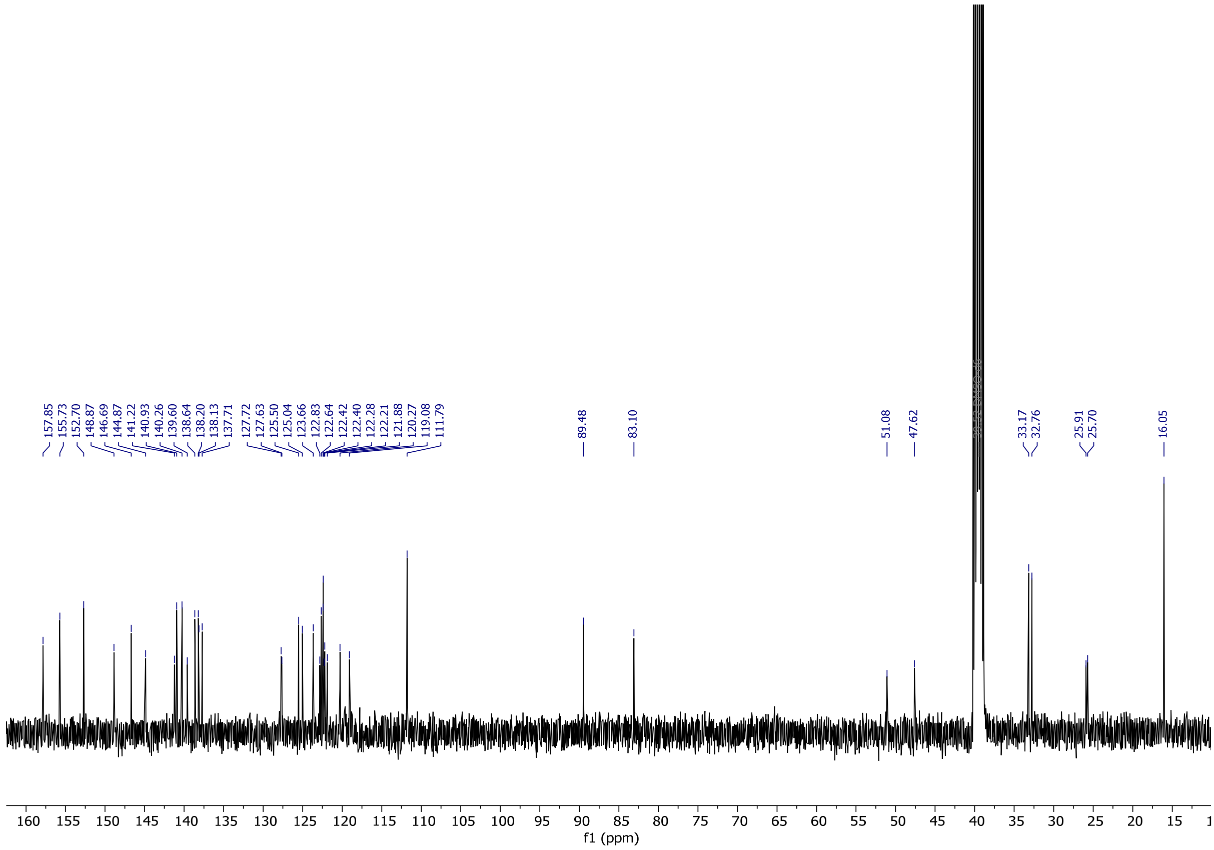
157.85
155.73
152.70
148.87
146.69
144.87
141.22
140.93
140.26
139.60
138.64
138.20
138.13
137.71
127.72
127.63
125.50
125.04
123.66
122.83
122.64
122.42
122.40
122.28
122.21
121.88
120.27
119.08
111.79
89.48
83.10
51.08
47.62
33.17
32.76
25.91
25.70
16.05
160 155 150 145 140 135 130 125 120 115 110 105 100 95 90 85 80 75 70 65 60 55 50 45 40 35 30 25 20 15 1
f1 (ppm)

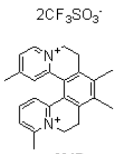

2CF3SO3-
N+
N+

MJB

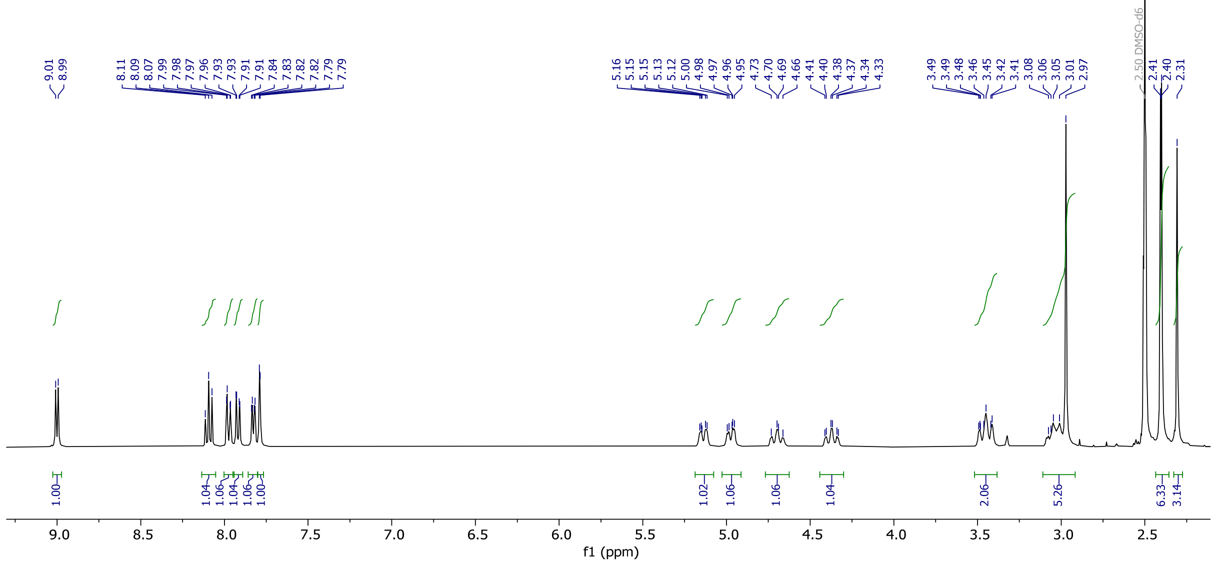

9.01
8.99
8.11
8.09
8.07
7.99
7.98
7.97
7.96
7.93
7.93
7.91
7.91
7.84
7.83
7.82
7.82
7.79
5.16
5.15
5.15
5.13
5.12
5.00
4.98
4.97
4.96
4.95
4.73
4.70
4.69
4.66
4.41
4.40
4.38
4.37
4.34
4.33
3.49
3.49
3.48
3.46
3.45
3.42
3.41
3.08
3.06
3.05
3.01
2.97
2.50 DMSO-d6
2.41
2.40
2.31
1.00
1.04
1.06
1.04
1.06
1.00
1.02
1.06
1.06
1.04
2.06
5.26
6.33
3.14
9.0 8.5 8.0 7.5 7.0 6.5 6.0 5.5 5.0 4.5 4.0 3.5 3.0 2.5
f1 (ppm)

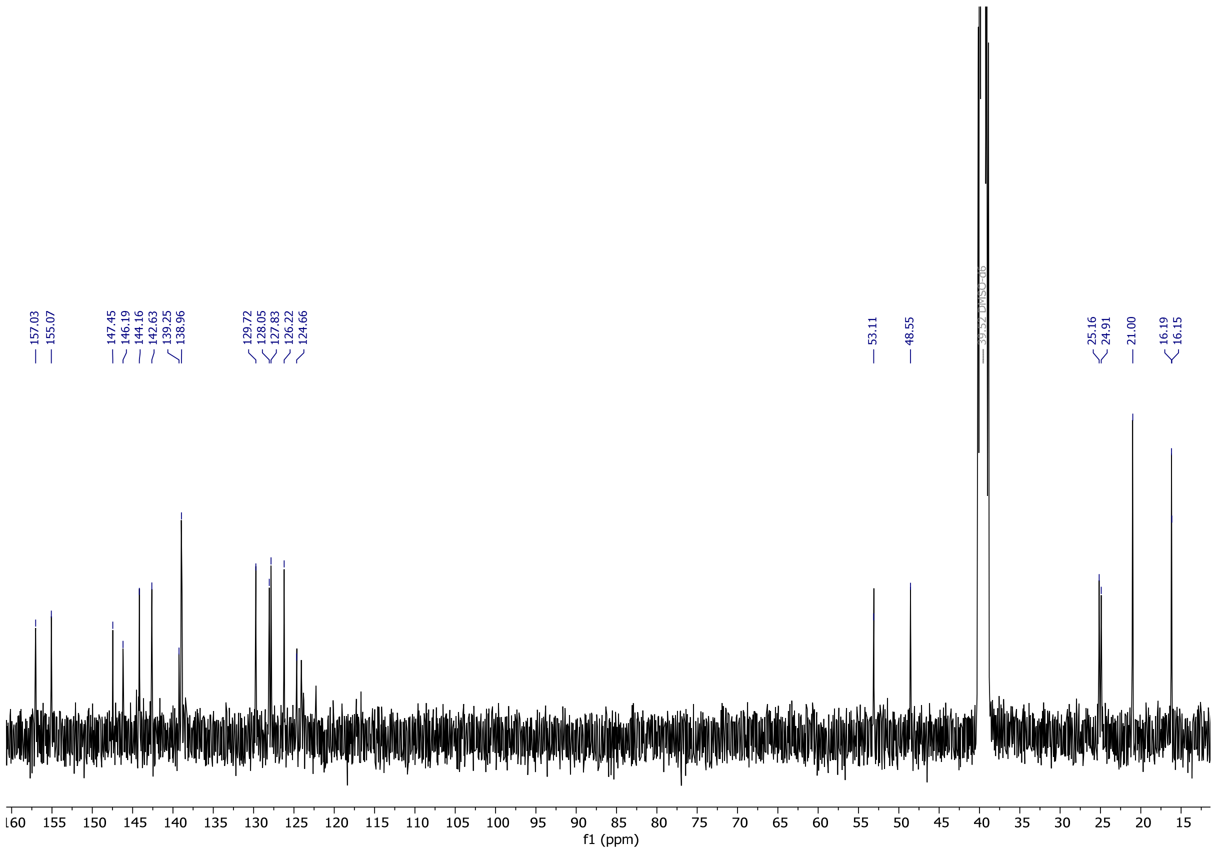

157.03
155.07
147.45
146.19
144.16
142.63
139.25
138.96
129.72
128.05
127.83
126.22
124.66
53.11
48.55
39.52 DMSO-d6
25.16
24.91
21.00
16.19
16.15
160 155 150 145 140 135 130 125 120 115 110 105 100 95 90 85 80 75 70 65 60 55 50 45 40 35 30 25 20 15
f1 (ppm)

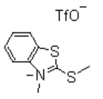
TfO⁻
S
N
S

PR739

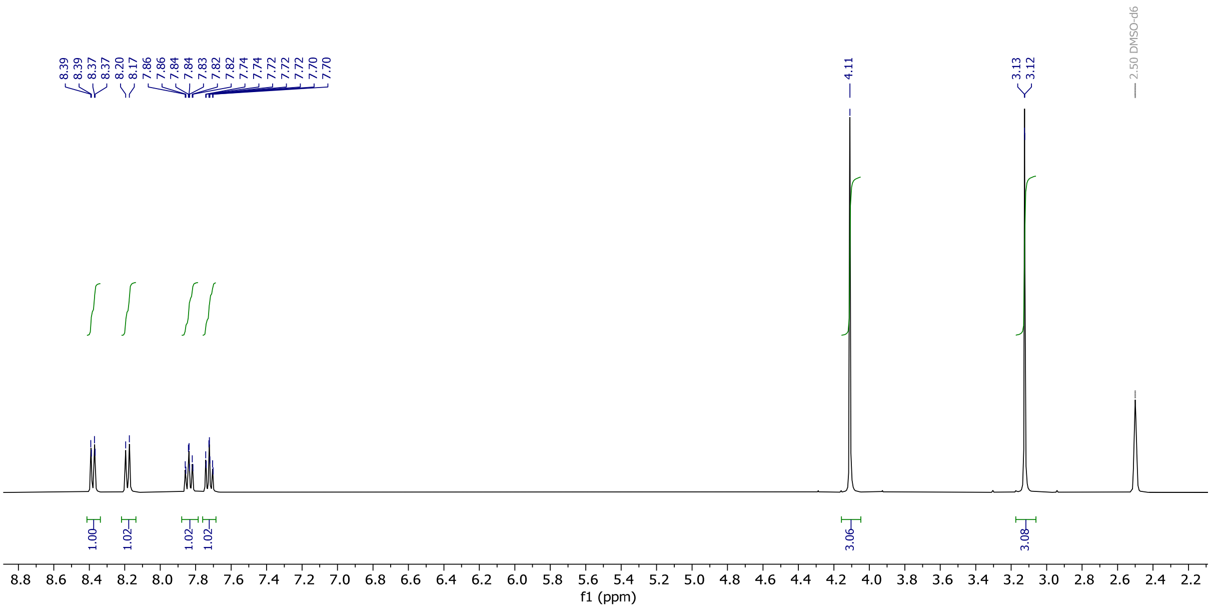
8.39 8.39 8.37 8.37 8.20 8.17 7.86 7.86 7.84 7.84 7.83 7.82 7.82 7.74 7.74 7.72 7.72 7.72 7.70 7.70
4.11
3.13 3.12
2.50 DMSO-d6
1.00 1.02 1.02 1.02 3.06 3.08
8.8 8.6 8.4 8.2 8.0 7.8 7.6 7.4 7.2 7.0 6.8 6.6 6.4 6.2 6.0 5.8 5.6 5.4 5.2 5.0 4.8 4.6 4.4 4.2 4.0 3.8 3.6 3.4 3.2 3.0 2.8 2.6 2.4 2.2
f1 (ppm)

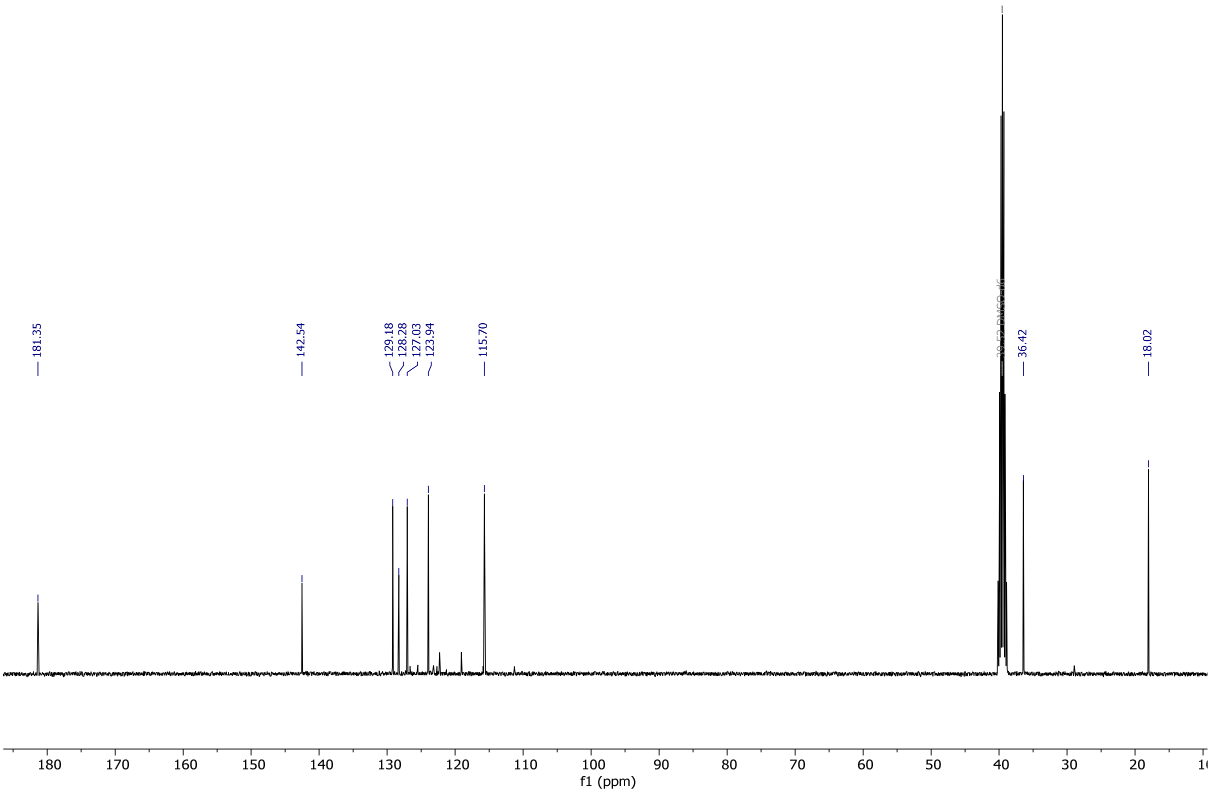
181.35
142.54
129.18 128.28 127.03 123.94
115.70
36.42
18.02
180 170 160 150 140 130 120 110 100 90 80 70 60 50 40 30 20
f1 (ppm)
